# Remarks on the Antagonism between Tubercular Phthisis and Marsh-Fever

**Published:** 1849-05

**Authors:** 


*Remarks on the Antagonism between Tubercular Phthisis and Marsh-fever.* By Dr. HELLFT, of Berlin. Translated from the German,* by DANIEL STAHL, M. D., of Quincy, Illinois.†

Many physicians, and among them, Boudin, especially, conclude from a vast number of facts, that phthisis has an antagonistic relation to marsh-fevers. The truth of this conclusion can only be tested by a statistical

---

* Schmiett's Jahrbücher der in-und ausländischen gesammten Medicin, 1848.—*Heft*, 4.

† Although the subject here treated of, is not new to the profession, yet as it is far from being well understood, and as it is of such vital importance, particularly to us in the United States, I thought that a leisure would not be illy spent in giving to the American medical public an abstract of the views of a German, who is well worthy of being heard. Whoever wishes more extensive information on the subject of the antagonism of diseases, particularly of phthisis and typhoid fever, with marsh fevers, etc., etc., would do well to consult "Etudes de Geologie Médicale sur la phthisie pulmonaire et la fièvre typhoid, dans leur Rapports avec les Localités Marécagensis, &c., &c. Par S. Ch. Boudin, Paris, 1845.—*Translator.*

examination and comparison of extensive observations, a method which, although attended with many difficulties, leads most safely to a decided result.

The author adds to the well known observations of Boudin and of several German physicians, those of a more recent date, and as the contradictory accounts might give rise to doubts touching the curative power of marsh miasm in phthisis, he shows the reasons why these observations differ from each other.

Levacher, for example, proves that in the marshy districts of the West Indies, where malaria generates intermittent and remittent fevers, phthisis is of frequent occurrence. Phthisis, according to Chisholm, is not a rare disease in those islands. Tschudi maintains, that intermittent fever and phthisis occur in equal proportion in Peru. In Brazil, where intermittents are the prevailing diseases, phthisis, according to Sigaud, kills one-fifth of the entire population, and of the colored portion, even the sixth or seventh part.

It is surprising, that despite of these facts, French physicians still recommend Algeria as a salutary residence for consumptive patients. Broussais invited the attention of phthisical sufferers to Algeria, and maintained that phthisis is not as frequent there as in France, ascribing this to the climate. He never found in the cadaveric examinations of those dead of other diseases, tubercles in the lungs; he does not deny, however, that cases of phthisis do occur there, although rarely, and that they develop themselves there, even during a prolonged residence near marshy localities, and after several attacks of fever. Bounafont, furthermore, makes mention of the fact, that during the winter and spring, pulmonary diseases prevail in Algiers, on account of the great changes of temperature; but he says in relation to the curability of pulmonary affections, that their cure, as far as they are curable, is very much aided by the temperature, which never sinks there as low as in northern countries.

The results, as to the influence of miasmatic effluvia on phthisical patients, are hence not the same every where. From all the accumulated observations, the author infers, that 1st, marsh miasmata, do not, in themselves, and alone, possess the curative or salutary power, but that with them, the degree of temperature, the equable temperature, the hygrometrical condition of the atmosphere, and lastly, the condition of the prevailing winds, must be taken into consideration. He therefore compares Gibraltar with Algeria. Notwithstanding the situation of both in the same degree of latitude, and the frequent occurrence in both places, of intermittent fevers, phthisis is more frequent in Gibraltar than in Algeria, on account of the great changes of temperature and the prevailing raw east winds.

2. That such an uniform atmosphere, impregnated with miasmatic effluvia, which is not too damp, affords protection only in the incipiency of the disease, or smothers the tubercular dyscrasia in its very bud.

A warm climate does not, as was formerly supposed, of itself, exercise any remedial influence on tubercular affections, but the uniformity of the temperature, the hygrometrical condition of the atmosphere and the prevailing winds do. As, for instance, on the Andes, where intermittent fevers prevail endemically, there often occur cases of phthisis, on account of the great changes of temperature, whereas, on the coast of British Guiana, phthisis is not known, because of the equableness of temperature.

An atmosphere which holds a medium between too great dryness and moisture, is most conducive to health. A residence at Hijeres, is, therefore, not as beneficial to the sufferer from phthisis, as was formerly supposed, because the air is too "sharp" and dry, from the frequency of northeasterly winds. Thus Casper found that at Berlin, at a time when the atmosphere possessed a certain degree of moisture, the least number of deaths from phthisis took place; the temperature, on the other hand, appeared to him to exercise but little influence. At Madeira, when no intermittent fevers are observed, phthisis is of rare occurrence, on account of the mild and even temperature of the atmosphere, with a proportionate moisture, as well at different seasons, as during the course of the day. For the same reason, Bullar found the climate of the Azores, where marsh-fevers are also rare, advantageous to the phthisical patient. Out of 465 who suffered from chronic diseases, he observed but two with phthisis. Confirmed phthisis is, however, not cured there, but rather hastened, as has been expressly remarked by Dr. Kämpfer in his notes on Madeira, and as has been shown by facts, by Heineken.

In order, however, to decide the question, the author thinks that not only the temperature and degree of moisture are of great importance, but also another remarkable antagonistical relation between phthisis and marsh-fever, in the different races of men, which also have different susceptibilities for endemic fevers. It has, for instance, been shown, that negroes have hardly any susceptibility for marsh-fevers, but often suffer from tubercles in the lungs. Tschudi says, that the Indians are most frequently affected with intermittents, next the whites, and last of all, the negroes and dark admixtures. The blacks on the Senegal, also, suffer chiefly from bronchitis and other pulmonary affections. A fifteenth part of all the natives die of such complaints, whilst of the whites, only 1.50 or even 1.100 die of the like. At Rio de Janeiro, according to Jobim, negroes rarely suffer from intermittents, whilst those born in Brazil, and the whites, are subject to them. According to Rufz, at Martinique, Europeans rarely, but most of mulattoes and negroes die of consumption. The accounts of the diseases, and the mortality in the English army from the year 1817 to 1836, show that of 1000 men, there died annually:

| | Of Intermittents. | | Of Pulmonary Affections. | |
|---|---|---|---|---|
| | Whites. | Blacks. | Whites. | Blacks. |
| In English Guiana,............ | 59.2 | 8.5 | 6.4 | 17.9 |
| At Trinidad,.................. | 61.6 | 3.2 | 11.5 | 16.4 |
| At Tabago,.................... | 104.1 | 8.6 | 11. | 12. |
| In Grenada,................... | 26.3 | 4.8 | 6.6 | 9.5 |
| At Barbadoes,................. | 11.8 | 3.8 | 15.8 | 18.7 |
| At St. Domingo,............... | 19.3 | 7.7 | 8.3 | 16.7 |
| At Jamaica,................... | 101.9 | 8.2 | 7.3 | 10.5 |
| In the Bahama Islands,........ | 159.1 | 5.6 | 6. | 9.7 |
| At Sierra Leon,............... | 410.2 | 2.4 | 6. | 6.3 |
| At the Mauritius,............. | 1.7 | 0.0 | 4. | 12.9 |
| At Ceylon..................... | 25.7 | 1.1 | 4.9 | 10.5 |

The immunity of negroes from marsh-fevers was also verified in the negro expedition of 1841. On three ships a malignant fever was developed, in spite of every precaution, so that of 145 individuals, 130 were taken sick, and 40 died. Of 158 blacks, there were only 11 slightly sick, and none died. these eleven having lived a long time in England. Major Tulloch remarks on the diseases of the Cape of Good Hope, that fevers are more rare among the Hottentots than among the whites, whereas, the former are more liable than the latter to pulmonary affections. Similar accounts are given by the French physicians of Algeria. There die, according to Guyon, many more native Moors and Jews, of phthisis, than Europeans, the latter succumbing more frequently to fevers.

The almost congenital immunity of natives from tubercles, in countries where marsh-fevers prevail endemically, is, according to the author, founded in a change of the organism, particularly of the blood, which manifests by a degeneration of the race of men who inhabit such countries, or of persons who are there acclimated. This view he believes to be sustained by the observation of Dr. Hille, of Surinam. The latter found, indeed, that the blood never showed a crusta inflammation, even during the most violent phlogistic symptoms. More than half the blood is serum, in which the crassamentum, as it were, swims. The latter is commonly dark red, while the serum is lighter than that of the Europeans. It is particularly remarkable, that the buffy coat is plainly discernable in the first inflammatory affections of those Europeans only, who have resided but a short time at Surinam; afterwards it disappears, and the blood has then all those appearances which are observed in that of negroes.

Pathological chemistry and all the recent anatomico-pathological researches have shown, that a high degree of serosity of the blood, *i. e.*, a diminution of the fibrin, excludes the possibility of the generation of tubercles. The production of tubercles requires an excess of fibrin in the blood, and certainly a qualitative, but as yet, unknown change in that fluid. Andral and Gavarret have already shown, that there is, from the incipiency of tubercular phthisis, a tendency to an increase of fibrin, and to a decrease in the number of blood corpuscles; that this change in the chemical composition of the blood is but slight, in the crude condition of the tubercles, but that it manifests itself plainly during the stage of softening. Becquesel and Rodier obtained the same results.

Engel expresses himself on this point, in the following words:—"In order to prevent a tubercle from any further change, to fence it off, as it were, from the organism, and to render it harmless to it, or to remove it out of it, it is necessary to put the organism in such a condition, as to render a repetition of the secretion of fibrin, and hence a re-production of tubercle impossible, or, as it is expressed, to efface the dyscrasia. During dropsical diseases and scurvy, or during such diseases as are akin to the latter, the production of tubercles cannot take place. Diseases with stagnation in the circulatory system, also protect against tubercles, such as liver and spleen affections, but then only when the above-mentioned condition, namely—increased serosity—takes place."

Now as all researches on the composition of the blood in intermittent fevers and their sequel, prove that the quantity of fibrin diminishes as these diseases prolong their course or recur, while the blood-corpuscles generally remain unchanged, it is probable that therein is to be found the cause why marsh-miasmata and the diseases dependent upon them, afford a kind of protection from the generation of tubercles.

Could, therefore, such individuals as have a predisposition to tubercles, be sent to countries where marsh-fevers are the prevailing maladies, at the same time taking the temperature and condition of the weather into consideration, the tubercular diathesis might be nipped in the bud. The above detailed condition of the blood is, however, always injurious in the advanced stage, or when softening of the tubercles has already occurred, because the ulcerations, now become atonic, cannot heal, and confirmed phthisis can, therefore, with such a blood, only be hastened in its destructive course.

The great influence of marsh-miasmata upon the generation of tubercles, manifests itself furthermore, in the continued immunity from phthisis, of such individuals as have resided in districts where marsh-fevers were endemical, long after they have left those districts. Thus it is told by Brunach, that at Marseilles, of 300 soldiers who have come from the marshy districts of Algeria and Corsica, only 2 were in the years of 1841–2 affected with phthisis, whilst among the inhabitants of Marseilles, 1 out of 4 deaths is from phthisis.—*St. Louis Medical and Surgical Journal.*

